# Neuroprotective Properties of Bis-Sulfonamide Derivatives Against 6-OHDA-Induced Parkinson's Model *via* Sirtuin 1 Activity and *in silico* Pharmacokinetic Properties

**DOI:** 10.3389/fnmol.2022.890838

**Published:** 2022-07-22

**Authors:** Setthawut Apiraksattayakul, Ratchanok Pingaew, Veda Prachayasittikul, Waralee Ruankham, Papitcha Jongwachirachai, Napat Songtawee, Wilasinee Suwanjang, Tanawut Tantimongcolwat, Supaluk Prachayasittikul, Virapong Prachayasittikul, Kamonrat Phopin

**Affiliations:** ^1^Center for Research and Innovation, Faculty of Medical Technology, Mahidol University, Bangkok, Thailand; ^2^Department of Chemistry, Faculty of Science, Srinakharinwirot University, Bangkok, Thailand; ^3^Center of Data Mining and Biomedical Informatics, Faculty of Medical Technology, Mahidol University, Bangkok, Thailand; ^4^Department of Clinical Chemistry, Faculty of Medical Technology, Mahidol University, Bangkok, Thailand; ^5^Department of Clinical Microbiology and Applied Technology, Faculty of Medical Technology, Mahidol University, Bangkok, Thailand

**Keywords:** bis-sulfonamide, 6-OHDA, Parkinson's disease, SIRT1, molecular docking, ADMET

## Abstract

Parkinson's disease (PD) is considered one of the health problems in the aging society. Due to the limitations of currently available drugs in preventing disease progression, the discovery of novel neuroprotective agents has been challenged. Sulfonamide and its derivatives were reported for several biological activities. Herein, a series of 17 bis-sulfonamide derivatives were initially tested for their neuroprotective potential and cytotoxicity against the 6-hydroxydopamine (6-OHDA)-induced neuronal death in SH-SY5Y cells. Subsequently, six compounds (i.e., **2, 4, 11, 14, 15**, and **17**) were selected for investigations on underlying mechanisms. The data demonstrated that the pretreatment of selected compounds (5 μM) can significantly restore the level of cell viability, protect against mitochondrial membrane dysfunction, decrease the activity of lactate dehydrogenase (LDH), decrease the intracellular oxidative stress, and enhance the activity of NAD-dependent deacetylase sirtuin-1 (SIRT1). Molecular docking was also performed to support that these compounds could act as SIRT1 activators. In addition, *in silico* pharmacokinetic and toxicity profile prediction was also conducted for guiding the potential development. Thus, the six neuroprotective bis-sulfonamides were highlighted as potential agents to be further developed for PD management.

## Introduction

Currently, the world is dealing with continual expansion of the elderly, in which 11% of the world's population aged over 60 years. This situation is estimated to be doubled to reach a hyper-aged population by the year 2050 (Kanasi et al., 2016). Accordingly, the prevalence of age-related diseases has continuously been increased, especially, the neurodegenerative disorders (Albers and Beal, 2000; Baker and Petersen, 2018). Neurodegenerative diseases are defined by progressive degenerations of the nervous system's structures that consequently leads to the impairments of multiple functions (i.e., memory loss, mental impairments, and physical abnormalities) (Gao and Hong, 2008; Alzheimer's, 2017). Neurodegenerative diseases are multifactorial diseases in which multiple factors play roles in their pathogenesis and progression. Oxidative stress is one of the critical factors leading to neurodegeneration. Oxidative stress is caused by the presence of excessive reactive oxygen species (ROS) accumulated in the cells. This condition subsequently induces the oxidative damages of cellular components (i.e., proteins, lipids, and DNA) leading to neurotoxicity and neuronal cell death (Emerit et al., 2004). Therefore, neurodegenerative diseases are considered global public health concerns due to their complexity and incurable nature. Out of which, Alzheimer's disease and Parkinson's disease (PD) are the two most common diseases found in the population.

The PD ranked as the second most prevalent neurodegenerative disorders, with the main characteristic of severe movement impairments. According to the report of World Health Organization (WHO), the PD is a neurological disorder with the fastest arising of worldwide new cases, disabilities, and fatalities among others (De Lau and Breteler, 2006; Dorsey et al., 2018). The severe movement impairments have been noted as a result of progressive destruction of the dopaminergic neurons in substantia nigra area leading to the reduction of endogenously synthesized dopamine (DA), which is a neurotransmitter that plays essential roles in the control of movement (Wood-Kaczmar et al., 2006). Similar to other neurodegenerative diseases, the exact causes of PD are still unknown; however, multiple factors (i.e., internal genetic factors and external environmental factors) are noted as possible risk factors. The genetic factors include the mutations of LRRK2, alpha-synuclein, parkin, DJ-1, PINK1, and other related genes, whereas the external factors are acquired by the exposure to toxic chemicals (i.e., paraquat) or some types of therapeutic drugs (i.e., aromatase inhibitors) as well as head trauma (Wood-Kaczmar et al., 2006; Kalia and Lang, 2015; Rosenfeld et al., 2018). The PD displays a unique pathological hallmark called Lewy bodies, which is an aggregation of several types of abnormal proteins within the brain cells. Among all, the alpha-synuclein, the most prominent one found in the Lewy bodies, has gained the most attention in the area of PD research (Rosenfeld et al., 2018). Unfortunately, the currently available drugs for PD treatment are only symptomatic drugs [i.e., levodopa, DA agonist, and monoamine oxidase B (MAO B) inhibitors] that can only alleviate the symptoms, but are incapable of preventing the progressive nature of the disease's progression and consequences (Hurtig, 1997; Potashkin and Seidl, 2011; Sarkar et al., 2016). Accordingly, the discovery and development of novel classes of neuroprotective agents with preventive potentials are considered an area of the urgent need for effective management of PD.

6-Hydroxydopamine (6-OHDA) is the first substance that has been identified as a dopaminergic neurotoxin. 6-OHDA is a DA analog with a high binding affinity to the DA transporter; therefore, it has a high potential to be selectively uptake into the catecholaminergic neurons, including the dopaminergic ones (Bové et al., 2005; Hernandez-Baltazar et al., 2017). Inside the neurons, the 6-OHDA undergoes enzymatic degradation facilitated by the monoamine oxidase-A (MAO-A) as well as the autoxidation. As a result, many cytotoxic compounds are generated as products of reactions leading to neuroinflammation. Furthermore, neurotoxicity of the 6-OHDA could be due to an ability of the compound to impair mitochondrial complex I and IV functions, leading to neurodegeneration and cell death in the PD (Glinka et al., 1997; Soto-Otero et al., 2000). According to its selectivity to target the dopaminergic neurons and abilities to replicate cellular processes and conditions implicated in the PD (i.e., oxidative stress and mitochondrial dysfunction), the 6-OHDA has been used in PD model for several decades (Aryal et al., 2020).

Sulfonamide, a sulfonyl group linked to an amino group, is one of the attractive pharmacophores in modern drug discovery and development due to its stability and ease of synthesis. The sulfonamide group is found in several types of therapeutic drugs [i.e., antihypertensive, antibacterial, and anti-inflammatory agents (Kołaczek et al., 2014)]. This compound is first known as an antimicrobial agent (Alsughayer et al., 2011; Ovung and Bhattacharyya, 2021). In addition, other biological activities of the sulfonamide-based compounds have been reported, including antiradical, antioxidant, and neuroprotective properties. Sulfonamide derivatives were reported as anti-Alzheimer agents with antioxidant properties along with other possible mechanisms relating to cellular protection (Göçer et al., 2013; Leechaisit et al., 2019; Peng et al., 2020; Gök et al., 2021). According to the literature, sulfonamide derivatives have the potential to be used in the treatment of neurological disorders due to their antioxidant properties, which are the fundamental mechanism of neuroprotection (Göçer et al., 2013; Lee et al., 2020; Peng et al., 2020). However, these findings have not been tested in human neural cells for neuroprotective purposes, and the underlying mechanism in neuronal cells is yet unknown. Therefore, the sulfonamide-based compounds are noteworthy to be investigated for their neuroprotective properties against the PD model of human neuronal cells as well as the underlying mechanisms to better understand how they affect neuronal functions.

Drug development is a complex time-consuming process with a low success rate. Most of the failures occur in the late stages due to poor pharmacokinetic profiles and toxicities. Computational *(in silico)* approaches have been widely used as facilitating tools for increasing success rate as well as saving time and cost in modern drug development (Shaker et al., 2021). Molecular docking is one of the most common tools used for understanding the possible binding modes and interactions of the investigated compounds with their molecular targets. *In silico* pharmacokinetic profile prediction has also been noted for prioritizing the potential drug-like compounds for further successful development (Wu et al., 2020).

**Figure 1:**
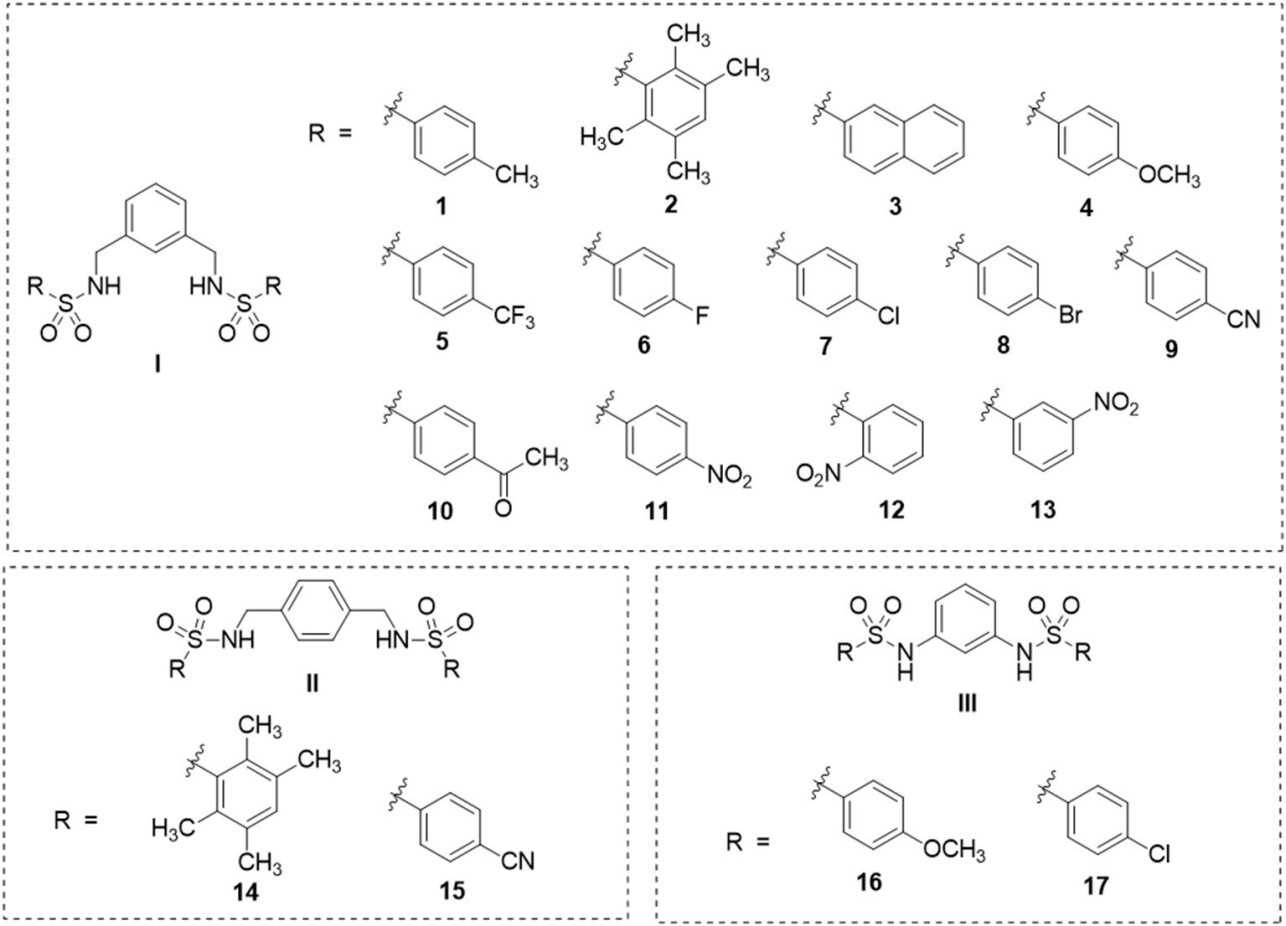
Chemical structures of in-house bis-sulfonamides (**1**–**17**).

In this study, a series of 17 bis-sulfonamide derivatives (**1**–**17**, Figure 1) previously reported by our group (Leechaisit et al., 2019) were investigated for their neuroprotective effects and underlying possible mechanisms against the 6-OHDA-induced neuronal cells death (i.e., cell viability, cell morphological changes, lactate dehydrogenase (LDH) activity, intracellular ROS, mitochondrial membrane potential (MMP), and NAD-dependent deacetylase sirtuin-1 (SIRT-1) activity). To elucidate the binding modes of actions and key interactions, molecular docking was performed. In addition, the pharmacokinetic profiles of the compounds were predicted to highlight their potentials for further development.

## Materials and Methods

### Chemicals and Reagents

Human neuroblastoma SH-SY5Y cell line (CRL-2266™) was obtained from American Type Culture Collection (Manassas, VA, USA.). Dulbecco's Modified Eagle Medium (DMEM) (Cat. No. 31600-034), fetal bovine serum (FBS) (Cat. No. 10270-106), 0.25% trypsin-ethylenediaminetetraacetic acid (EDTA) (Cat. No. 25200-072) penicillin, and streptomycin (Cat. No.15140-122) were purchased from Gibco BRL (Gaithersburg, MD, USA). 3-(4,5-Dimethylthiazol-2-yl)-2,5-diphenyltetrazolium bromide (MTT) (Cat. No. M6494) and cell-permeant 2′,7′-dichlorodihydrofluorescein diacetate (H_2_DCFDA) (Cat. No. D399) were obtained from Molecular Probes (Eugene, Oregon, USA). LDH-Activity Assay Kit (Cat. No. MAK066), Sirt-1 Activity Assay Kit (Cat. No. CS1040-1KT), mitochondrial-specific fluorescent dye (rhodamine-123) (Cat. No. R8004), 6-OHDA (Cat. No. H4381), and resveratrol (R5010-100MG) were obtained from Sigma Aldrich (St Louis, MO, USA).

### Cell Culture

The human neuroblastoma (SH-SY5Y) cells were cultivated with DMEM supplement containing 10% heat-inactivated FBS and 1% penicillin/streptomycin in 75-cm^2^ culture flasks. The cells were maintained at 37°C in a humidified air incubator with 95% air and 5% CO_2_. The culture medium was replaced every 3 days, and the cells were passaged after confluence reached 80% (Gay et al., 2018).

### Measurement of Cell Viability by MTT Assay

The MTT (3-(4,5-dimethylthiazol-2-yl)-2,5-diphenyltetrazolium bromide) assay is a colorimetric technique for determining cell metabolic activity as a viability indicator. In this experiment, the SH-SY5Y cell line (1 × 10^5^ cells/mL) was cultivated in 96-well plates for 24 h. The plates were then incubated for 3 h with various doses of bis-sulfonamides or resveratrol (0.1–100 μM), followed by another 24 h with 100 μM of 6-OHDA (Jing et al., 2015). Then, 5 mg/mL of MTT solution was added to each well, followed by a 2–4 h incubation at 37°C in the dark. For active cells, yellow tetrazolium salt (MTT) is converted into insoluble purple formazan crystals by dehydrogenase enzymes. At the termination of the incubation period, the solution was withdrawn, and 0.04 N HCl in isopropanol was added to solubilize the formazan crystals, producing a purple color solution measured by using a microplate reader at 570 nm. The absorbance of formazan is directly proportional to the number of viable cells. The viability of the cells was stated as the percentage relative to the control group's viability (Gay et al., 2018).

### Cell Morphology Assessment

SH-SY5Y cells, at a density of 1 × 10^5^ cells/mL, were cultured on a cell culture petri dish overnight, allowing them to adhere. After seeding, the cells were pretreated with bis-sulfonamides for 3 h and exposed to 100 μM of 6-OHDA for another 24 h. At the end of the incubation period, an inverted light microscope (Olympus Corporation, Tokyo, Japan) at 20× magnification was used to observe the morphology of the cells. A digital camera was used to capture the photos of each treated and untreated cell from numerous distinct fields (Pratiwi et al., 2021).

### Determination of Cell Membrane Damage by LDH Assay

The LDH leakage into the culture medium was used to assess the compound's neuroprotective effect against 6-OHDA-induced cell cytotoxicity. SH-SY5Y cells (2 mL) at a density of 1.0 × 10^5^ cells/ml were cultured overnight in 6-well plates to prepare samples for the LDH test. The plates were then pretreated with 5 μM of bis-sulfonamides or resveratrol for 3 h before being exposed to 100 μM of 6-OHDA for another 24 h. Following that, the culture medium was collected, and LDH activity was evaluated using the LDH assay kit following the manufacturer's protocol from Sigma Aldrich (St Louis, MO, USA.). The assay is based on the conversion of lactate to pyruvate, which generates NADH, resulting in a change in absorbance at 450 nm. The LDH was expressed as the percentage relative to the control group.

### Detection of Intracellular ROS Production

Intracellular ROS production was indicated by using the ROS-sensitive fluorescent probe [carboxy-dichlorofluorescin diacetate (DCFDA)]. The cells were plated overnight at a concentration of 1.0 × 10^5^ cells/mL in 96-well plates followed by pretreating with bis-sulfonamides for 3 h and 6-OHDA for another 24 h. Following that, carboxy-H_2_DCFDA was added at a final concentration of 10 μM, and plates were incubated for 30 min in the dark. ROS levels were detected using a microplate reader at excitation and emission spectra of 495 and 527 nm, respectively (Sooknual et al., 2020).

### Measurement of Mitochondrial Membrane Potential by Rhodamine-123

The MMP was determined by mitochondrial-specific fluorescent dye, rhodamine-123. The cells were plated overnight at a concentration of 1.0 × 10^5^ cells/mL in each well of 96-well plates followed by pretreatment with bis-sulfonamides for 3 h and exposure to 100 μM of 6-OHDA for another 24 h. Following that, rhodamine-123 was added at a final concentration of 10 μM, and plates were incubated for 30 min in the dark. After the incubation, cells were washed twice with phosphate buffered saline (PBS), and MMP levels were detected by a microplate reader at excitation and emission wavelengths of 488 and 525 nm (Sooknual et al., 2020).

### Measurement of SIRT1 Activity

SIRT-1 deacetylase activity was determined using the SIRT1 assay kit from Sigma Aldrich (St Louis, MO, USA), performed under the manufacturer's instructions. In addition, SH-SY5Y cells were seeded on 6-well plate overnight followed by pretreating with bis-sulfonamides for 3 h and another 24 h exposure to 6-OHDA. The cells were rinsed with ice-cold PBS and extracted in a lysis buffer, including protease inhibitors at 4°C for 20 min. Next, the cells were scraped from the culture dish, followed by 12,000 × *g* centrifugation for 20 min at 4°C. Bradford protein assay (Bio-Rad Laboratories, Hercules, CA, USA) was performed following the manufacturer's protocol to measure the concentration of protein in the samples. The extracted proteins were investigated for SIRT1 activity following the manufacturer's protocol. The fluorescence intensity was measured at excitation wavelength of 340 nm and emission wavelength of 445 nm with a fluorescent microplate reader. SIRT1 activity was calculated as the percentage relative to the control.

### Prediction of Pharmacokinetic Properties

The web-based tools, including SwissADME (http://www.swissadme.ch/), pkCSM servers (http://biosig.unimelb.edu.au/pkcsm/), and ProTox-II [ProTox-II—Prediction of TOXicity of chemicals (charite.de)], were used to predict pharmacokinetic parameters of the studied compounds. The compounds structures in SMILES format were uploaded on the servers to predict their physicochemical characteristics, pharmacokinetic properties (i.e., absorption, distribution, metabolism, and elimination [ADME]), and toxicity profiles were calculated. Finally, Lipinski's rule of five (LRo5) and Veber's rule were used to evaluate the drug-likeness of the compounds.

### Molecular Docking

Molecular docking was performed *via* AutoDockTools version 4.2.6 to investigate possible binding modes of a selected compound against the NAD-dependent deacetylase SIRT1 (Morris et al., 2009). Crystallographic structure of an activated state human SIRT1 in complex with co-crystallized ligands (i.e., three molecules of resveratrol) and an AMC-containing peptide at 3.2 Å resolution was obtained from the Protein Data Bank (PDB code 5BTR; Cao et al., 2015). Prior to docking, the co-crystalized ligands were subtracted from the target protein, and only chain A was selected for docking. The protein was subsequently prepared by adding the polar hydrogen atoms and charges using the AutoDockTools. The following docking parameters were used: grid box size (50^*^50^*^50 points) and grid spacing of 0.375 Å. The grid box's center was placed at −23.315, 65.890, and 14.723 to ensure that the whole interface between the C-terminal domain (CTD) and the N-terminal domain (NTD) of the SIRT-1, which is an activator-binding site, was covered. Protein structure's rotational bonds were defined as rigid, while compound structure's rotational bonds were considered flexible. For each docking simulation, the number of independent docking runs was set at 100 runs, and the search parameter was the Lamarckian Genetic Algorithm, in which the maximum number of energy was adjusted to the medium level (Morris et al., 1998). Validation of the reliability of the docking protocol was accomplished by computing the root-mean-square deviation (RMSD) to determine the structural difference between the co-crystallized resveratrol and redocked resveratrol molecules. After the docking process, the binding interaction between SIRT-1 and investigated compounds was analyzed and visualized using the Discovery Studio Visualizer 2016 (BIOVIA, LLC; Pratiwi et al., 2021).

### Statistical Analysis

The results were expressed as the mean ± SEM. Statistical comparisons between groups were performed by one-way analysis of variance (ANOVA) followed by a Tukey–Kramer *post-hoc* test using the GraphPad Prism 6 scientific software (Graph Pad Software, Inc., La Jolla, CA 92037, USA). A value of probability (*P*) <0.05 was considered to be statistically significant.

## Results

### Chemistry

Three sets of bis-sulfonamide derivatives (set I, **1**–**13**; set II, **14**–**15**; set III, **16**–**17**) bearing various substituents (R) on sulfonyl moieties (Figure 1) were synthesized *via N*-sulfonation of diamines and benzenesulfonyl chlorides reported by our group (Leechaisit et al., 2019).

### Effect of the Compounds (1–17) on Neuronal Cell Viability

**Figure 2:**
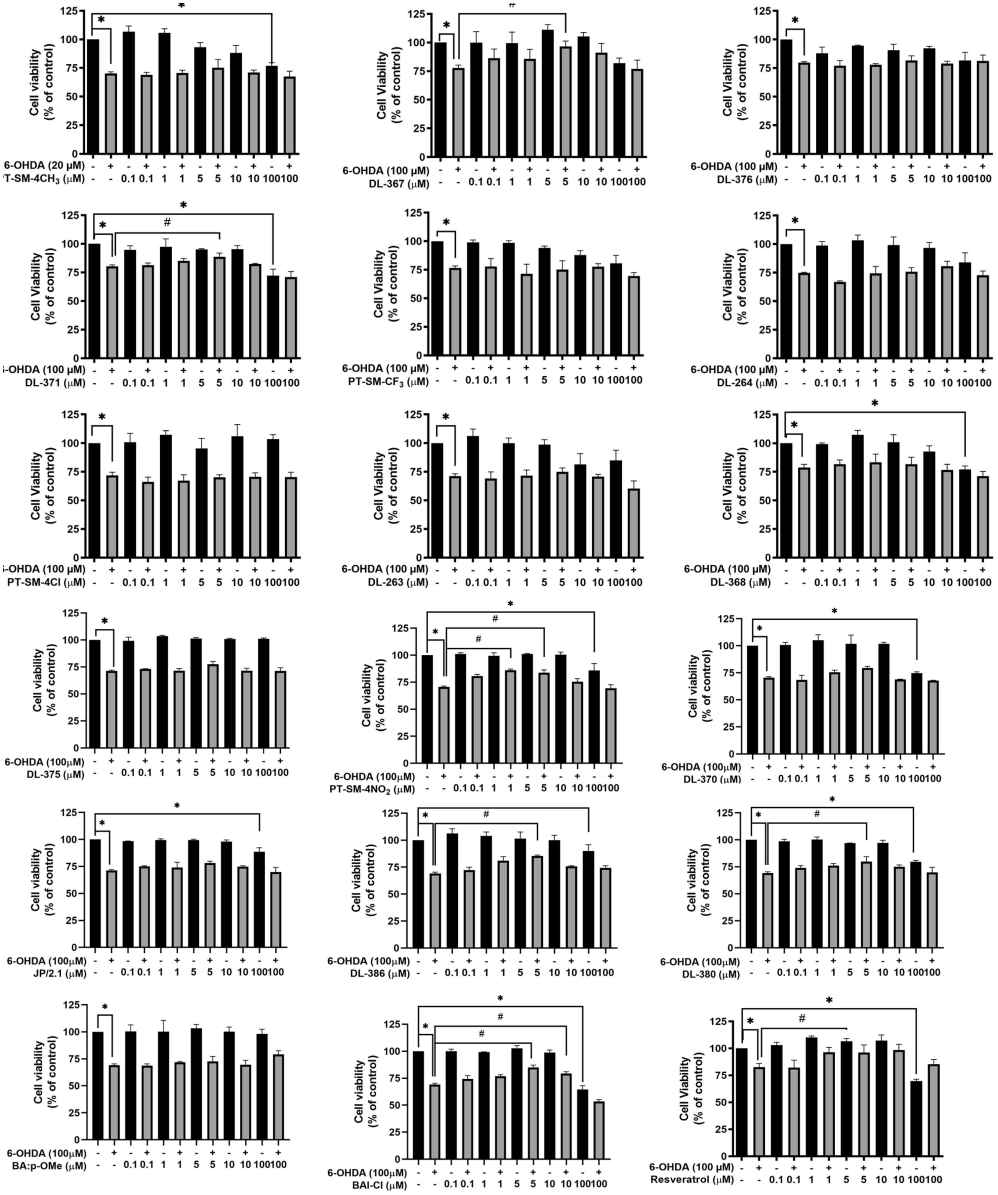
Bis-sulfonamides were studied in neuroblastoma SH-SY5Y cells for their neuroprotective effects. The SH-SY5Y cells (1 × 10^5^ cells/ml) were cultivated in 96-well plates for 24 h. The plates were then incubated for 3 h with various doses of bis-sulfonamides or resveratrol (0.1–100 μM), followed by another 24 h with 100 μM of 6-hydroxydopamine (6-OHDA) and evaluated by MTT assay. The data are expressed as mean ± SEM, and statistical analysis was performed using one-way ANOVA. *P < 0.05 compared with the control; ^#^*P* < 0.05 compared with the 6-OHDA.

The effects of bis-sulfonamide derivatives (**1**–**17**) on the viability of neuroblastoma SH-SY5Y cells were determined using the MTT assay. Oxidopamine, also known as 6-OHDA, was used as a neurotoxic agent to mimic the PD model. According to the preliminary report, 6-OHDA at 100 μM was used to develop Parkinson's model in SH-SY5Y cells and found to drastically reduce cell viability down to 70%, and other results also revealed distinct differences between the treated and untreated cells. Therefore, 6-OHDA at a concentration of 100 μM was selected to stimulate Parkinson's model in this study (Jing et al., 2015). SH-SY5Y cells were initially pretreated with different concentrations (varying from 0.1 to 100 μM) of the bis-sulfonamide derivatives in the absence of 6-OHDA for 24 h. No significant influence on the cell viability was observed for the tested bis-sulfonamides at the concentrations of 0.1, 1, 5, and 10 μM. Somehow, some derivatives significantly reduced the cell viability to 65% when compared to the control at high concentration (100 μM). It was also evident that the SH-SY5Y cells treated with 100 μM 6-OHDA alone for 24 h significantly decreased the cell viability by ~30%, as shown in Figure 2.

In addition, neuroprotective effects of the tested compounds were studied in the SH-SY5Y cells by pretreating the cells with each compound for 3 h before exposing them to 100 μM 6-OHDA for an additional 24 h. The results showed that significant recovery of the cell viability up to 79–96%, when compared to 6-OHDA alone, was observed for the cells pretreated with compounds DL-367 (**2**), DL-371 (**4**), PT-SM-4NO2 (**11**), DL-386 (**14**), DL-380 (**15**), and BA1-CL (**17**) at a concentration of 5 μM (Figure 2). Therefore, these six compounds (i.e., **2**, **4**, **11**, **14**, **15**, and **17**) at a concentration of 5 μM were selected for further experimental investigations to reveal possible mechanisms of neuroprotection.

### Effect of the Selected Compounds on Morphological Changes of the Cells

**Figure 3:**
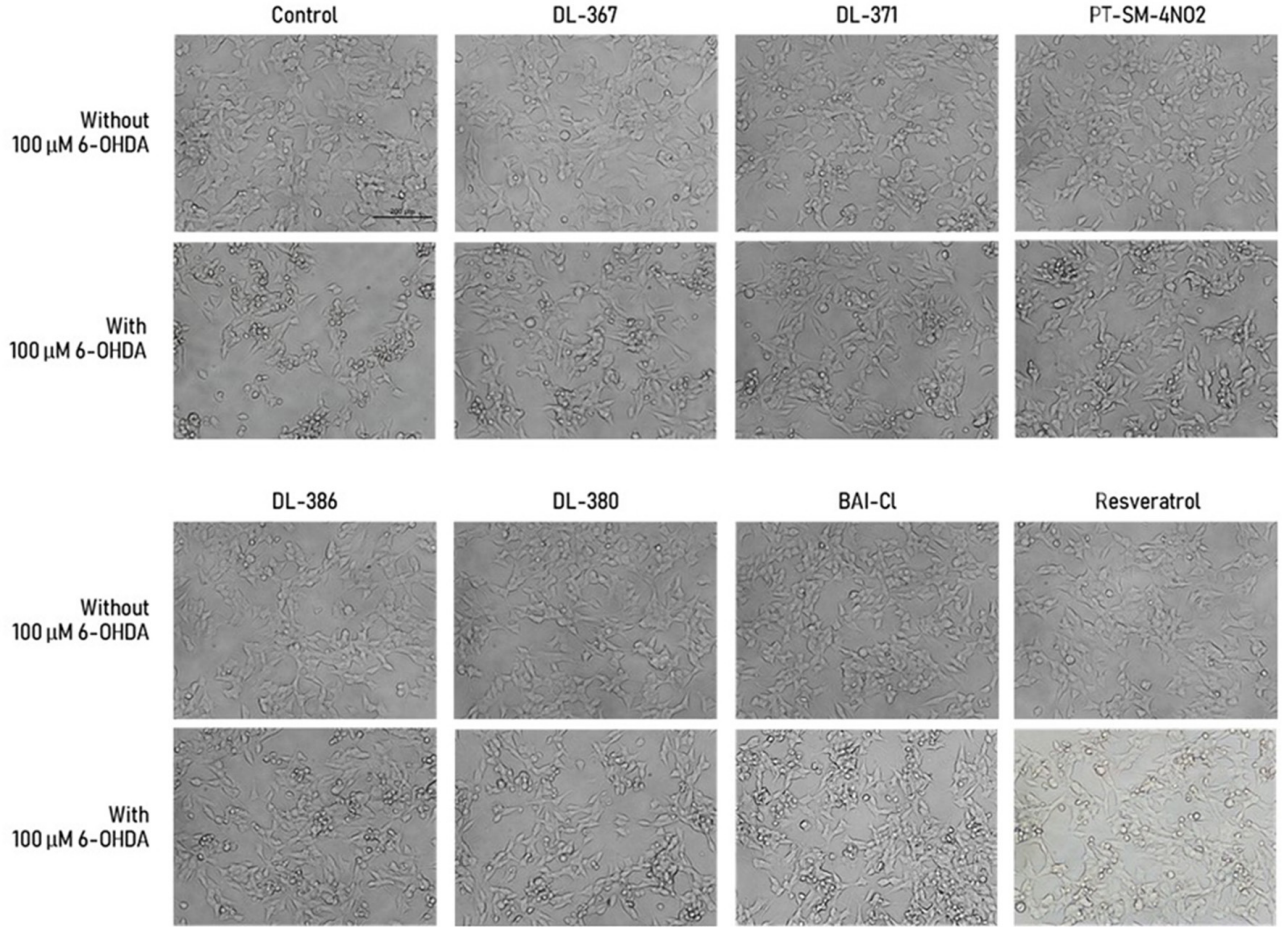
Morphological alteration of SH-SY5Y cells was observed under the inverted-light microscope at 20× magnification. Scale bar = 200 μm.

Morphological observation of the cell was performed under bright field microscopy to further investigate the protective effects of the selected compounds (i.e., **2**, **4**, **11**, **14**, **15**, and **17**) on SH-SY5Y cells. Morphological alterations were obviously observed for the cells treated with the toxic 100 μM 6-OHDA when compared to the control as shown by shrinkage and unattachment of the cells as well as the change of cell shape into a small round shape (Figure 3). For the cells pretreated with the selected sulfonamides or resveratrol (as positive control) at 5 μM for 3 h before exposure with the 6-OHDA, the minimized aberrant morphological changes were observed, and the cells displayed less destruction as well as more intact appearance and cell growth with adequate cell confluence when compared to those without the pretreatment before exposure to the 6-OHDA (Figure 3). This suggested that the pretreatment of the selected compounds could prevent the aberrant morphological changes in the cells.

### Effect of the Selected Compounds on Neuronal Damages and LDH Activity

**Figure 4:**
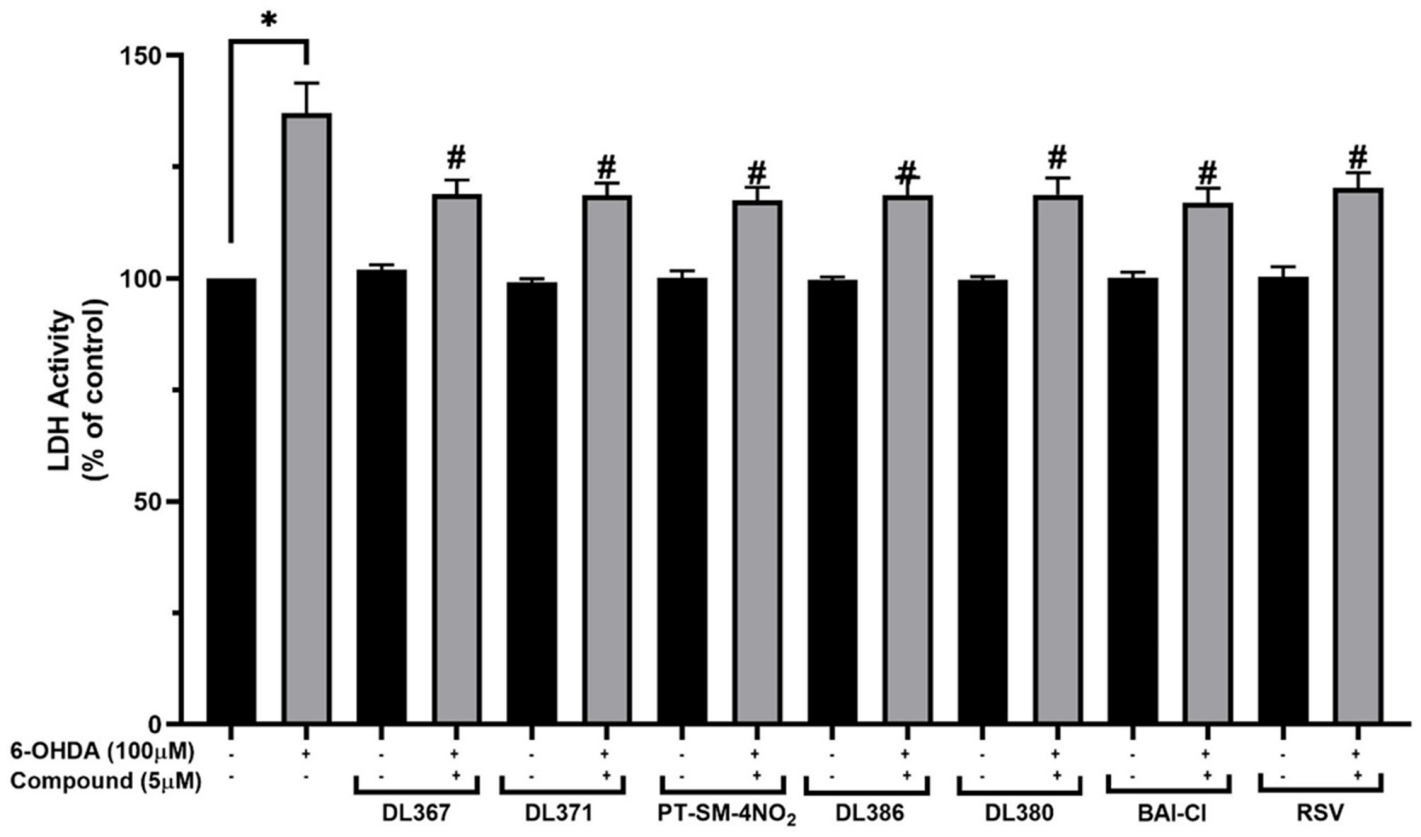
Measurement of lactate dehydrogenase (LDH) levels in culture medium. The SH-SY5Y cells were incubated with 5 μM of bis-sulfonamides or resveratrol for 3 h followed by 100 μM of 6-OHDA for 24 h. Then, the culture medium was collected and determined by LDH assay. The data are expressed as mean ± SEM, and statistical analysis was performed using one-way ANOVA. *P < 0.05 compared with the control cells; ^#^*P* < 0.05 compared with the 6-OHDA.

The LDH is an enzyme indicator for cellular damages in which the LDH activity will be increased in the conditions with cell membrane leakage and rupture. The selected bis-sulfonamides, i.e., **2**, **4**, **11**, **14**, **15**, and **17** were subsequently evaluated for their effects on LDH activity in the culture medium. It was observed that the LDH activity was increased by 137.16 ± 6.7% in the cells exposed to the neurotoxic 6-OHDA when compared to the control group (Figure 4). In contrast, the decreased LDH activity was observed for the cells pretreated with 5 μM of bis-sulfonamides as well as those of resveratrol (116.94 ± 3.3% to 118.91 ± 3.2%) when compared to the control group (Figure 4).

### Effect of the Selected Compounds on Intracellular ROS Production

**Figure 5:**
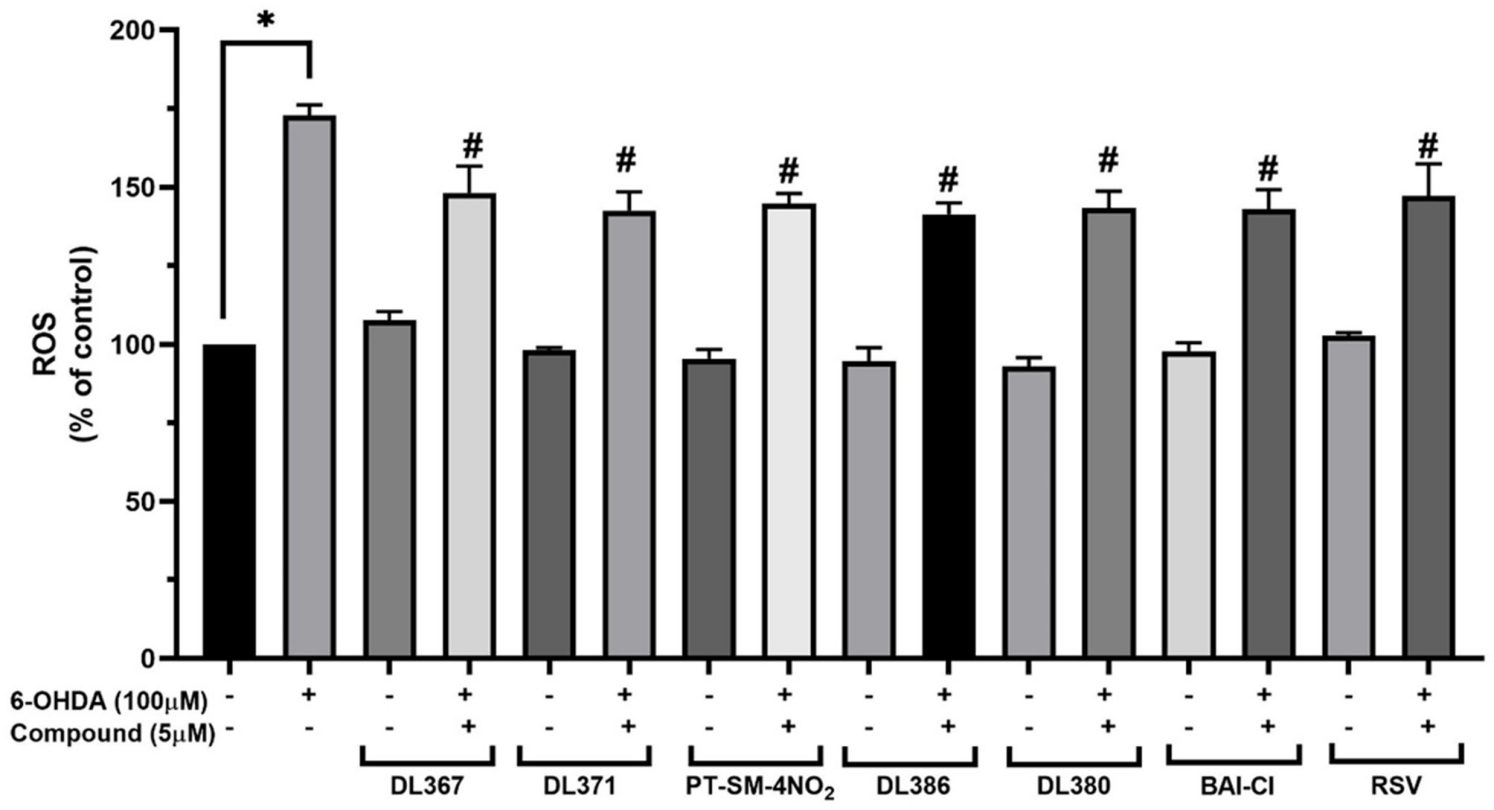
Measurement of intracellular reactive oxygen species (ROS) levels in SH-SY5Y cells on 6-OHDA-induced ROS generation. The SH-SY5Y cells were incubated with 5 μM of bis-sulfonamides or resveratrol for 3 h followed by 100 μM of 6-OHDA for 24 h and determined by carboxy-DCFDA assay. The data are expressed as mean ± SEM, and statistical analysis was performed using one-way ANOVA. *P < 0.05 compared with the control cells; ^#^*P* < 0.05 compared with the 6-OHDA.

The effect of the selected compounds, i.e., **2**, **4**, **11**, **14**, **15**, and **17** on intracellular ROS levels was investigated using a fluorescence probe, DCFDA, to reveal their antioxidant capacities. A significant increase in the fluorescence intensity up to 172.89% ± 3.3 was observed for the cells exposed to 100 μM of 6-OHDA for 24 h when compared to the control group (Figure 5). However, the pretreatment of the cells with 5 μM of the tested compounds or resveratrol for 3 h before exposure to the 6-OHDA can considerably reduce the intracellular ROS accumulation down to the range of 141.3 ± 3.7 or 148.1 ± 8.6% when compared to the control group (Figure 5).

### Effect of the Selected Compounds on Mitochondrial Membrane Potential

**Figure 6:**
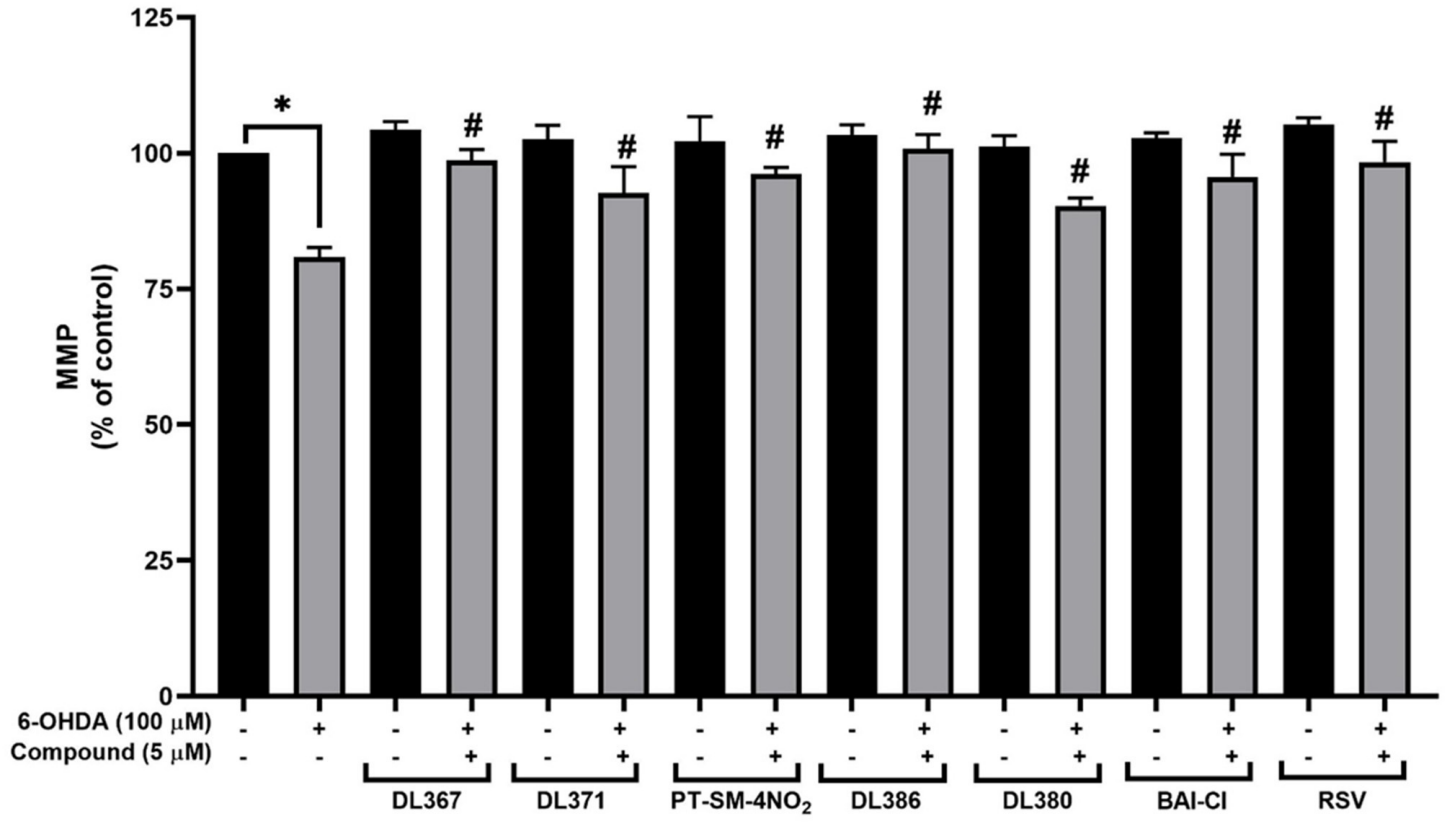
Effects of 5 μM resveratrol and bis-sulfonamides pretreatment for 3 h before exposure to 100 μM 6-OHDA for 24 h of SH-SY5Y cells on the mitochondrial membrane potential (MMP) as measured by the rhodamine-123 fluorescence intensity. The data are expressed as mean ± SEM, and statistical analysis was performed using one-way ANOVA. *P < 0.05 compared with the control cells; ^#^*P* < 0.05 compared with the 6-OHDA.

Mitochondria play vital roles in energy production, and its dysfunction has been noted in the pathogenesis of the PD (Winklhofer and Haass, 2010; Bose and Beal, 2016). MMP has been used to represent the mitochondrial functions, in which the decreased MMP indicates mitochondrial dysfunction and early stages of apoptosis (Brunelle and Letai, 2009; Martinou et al., 2011; Zhu and Xia, 2016; Cenini et al., 2019). To further investigate the neuroprotective action of the selected compounds (i.e., **2**, **4**, **11**, **14**, **15**, and **17**), an investigation of MMP was performed using the mitochondrial-specific fluorescent dye, rhodamine-123. The assay was performed by detecting the fluorescence signal from the accumulated dye within the functional membrane based on the membrane electrochemical gradient, in which the decreased fluorescent signal indicated the decrease of membrane potential (Ferlini and Scambia, 2007). For the cells exposed to 100 μM of toxic 6-OHDA for 24 h alone, the percentage of MMP was dramatically decreased to 80.84 ± 1.78% when compared to the control group (Figure 6). In contrast, the percentage of MMP was found to be notably improved (MMP: 90.33 ± 1.38 and 100.84 ± 2.57%) for the cells pretreated with 5 μM of the selected compounds and resveratrol for 3 h (Figure 6). This indicated that the pretreatment with the tested compounds could protect against 6-ODHA-induced mitochondrial dysfunction.

### Effects of the Compounds on SIRT-1 Activity

**Figure 7:**
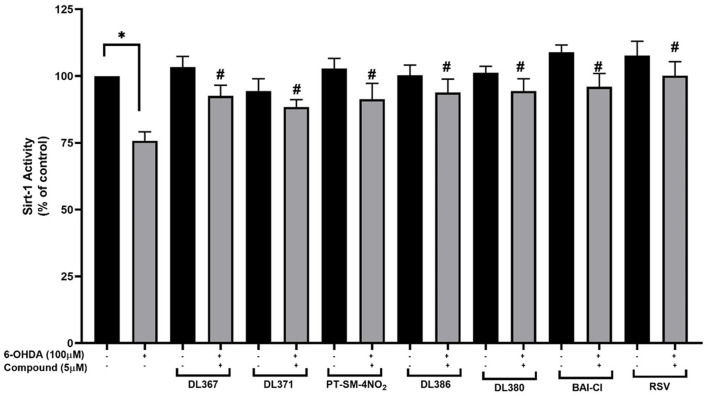
Measurement of SIRT-1 activity. The SH-SY5Y cells were incubated with 5 μM of bis-sulfonamides or resveratrol for 3 h followed by 100 μM of 6-OHDA for 24 h; the cells were extracted in a lysis buffer and scraped from the culture dish followed by centrifugation. The supernatant was collected and determined protein concentration by the Bradford assay and investigated further for SIRT-1 activity. The data are expressed as mean ± SEM, and statistical analysis was performed using one-way ANOVA. *P < 0.05 compared with the control cells; ^#^*P* < 0.05 compared with the 6-OHDA.

The selected compounds were investigated for their effects on SIRT-1 activity in the SH-SY5Y cells. Results showed that the SIRT-1 activity was significantly reduced to 75% in the 6-OHDA-treated cells after 24 h of incubation when compared to control cells (Figure 7). Pretreatment with the selected compounds for 3 h before 6-OHDA exposure showed the maintained activity of SIRT1 within the high range of 96% when compared to the control group. However, the studied compounds were less effective than the resveratrol, a known SIRT-1 activator, which displayed the complete maintenance of activity up to 100% (Figure 7). This suggested that the tested compounds could restore the activity of SIRT-1.

### *In silico* Prediction of Pharmacokinetic Properties of the Selected Compounds

*In silico* web-based tools were used to predict pharmacokinetic properties (i.e., SwissADME and pkCSM) and toxicities (i.e., ProTox) of the six selected compounds (i.e., **2**, **4**, **11**, **14**, **15**, and **17**). A set of physicochemical properties are revealed in Table 1. Results showed that all investigated compounds were estimated to have ideal lipophilicity for oral and intestinal absorption (ilogP value <5). This could be due to R groups of the compounds (Figure 1) containing hydrophobic phenyl ring with various substituents. Moreover, all compounds exhibited poor water solubility, except for DL-371 (**4**) as resulting from its polar OCH_3_ group. All selected compounds displayed high human intestinal absorption percentages (72.86–91.34%), indicating their potentials to be used as oral drugs. Most of the investigated compounds demonstrated moderate permeability across the blood–brain barrier (BBB) which suggests the possibility to reach the target site in the brain. However, the PT-SM-4NO2 (**11**) showed low BBB permeability which might be due to the ionic character of the NO_2_ group containing in its molecule. The cytochrome P450 family plays essential roles in the metabolism and detoxification of many drugs and substances. Among all, CYP2D6 and CYP3A4 are two subtypes responsible for the metabolism of more than 90% of drugs (Lynch and Price, 2007). Results indicated that all compounds were substrates of the CYP2D6 which implies their abilities to be metabolized in the liver. In addition, all selected compounds showed a preferable safety profile as predicted by none of hepatotoxicity, immunogenicity, cytotoxicity, carcinogenicity, and mutagenicity as well as their relatively high lethal dose (LD_50_). For the drug-likeness of the compounds, all compounds pass the criteria of both Lipinski's and Veber's rules, except for the PT-SM-4NO2 (**11**) which violated both of rules.

**Table 1:** Predicted^#^ physicochemical characteristics, pharmacokinetic and toxicity profile, and drug-likeness of the selected bis-sulfonamides (**2**, **4**, **11**, **14**, **15**, and **17**)^#^.

Compound^*^	**2**	**4**	**11**	**14**	**15**	**17**
**Physicochemical properties**						
MW^a^ (g/mol)	528.73	476.57	506.51	528.73	466.53	457.35
Rotatable bonds	8	10	10	8	8	6
H-bond acceptors	6	8	10	6	8	4
H-bond donors	2	2	2	2	2	2
TPSA^b^	109.10	127.56	200.74	109.1	156.68	109.1
**Absorption**						
Lipophilicity (iLOGP)	4.10	2.74	1.38	4.19	2.53	2.42
Water solubility	Poor	Moderate	Poor	Poor	Poor	Poor
GI absorption (% absorbed)	86.017	72.869	77.511	84.997	76.414	91.344
**Distribution**						
BBB^c^ permeant (log BB)	−0.205	−0.78	−1.497	−0.217	−0.619	−0.33
**Metabolism**						
CYP2D6 substrate	No	No	No	No	No	No
CYP3A4 substrate	Yes	Yes	Yes	Yes	Yes	Yes
**Excretion**						
Total clearance (log mL/min/kg)	1.152	1.106	0.999	0.907	1.227	−0.344
**Toxicity**						
Hepatotoxicity	No	No	No	No	No	No
Carcinogenicity	No	No	No	No	No	No
Immunotoxicity	No	No	No	No	No	No
Mutagenicity	No	No	No	No	No	No
Cytotoxicity	No	No	No	No	No	No
Predicted ${\text{LD}}_{50}^{\text{d}}$ (mg/kg)	7,900	2,800	4,470	7,900	2,250	2,500
**Drug-likeness**						
Lipinski's rule	Yes	Yes	No	Yes	Yes	Yes
Veber's rule	Yes	Yes	No	Yes	No	Yes

# *The prediction was performed via SwissADME (http://www.swissadme.ch/), pkCSM servers (http://biosig.unimelb.edu.au/pkcsm/), and ProTox-II [ProTox-II - Prediction of TOXicity of chemicals (charite.de)]*.

* *DL-367 (**2**), DL-371 (**4**), PT-SM-4NO2 (**11**), DL-386 (**14**), DL-380 (**15**), and BA1-CL (**17**)*.

a *MW, molecular weight*.

b *TPSA, total polar surface area*.

c *BBB, blood-brain-barrier*.

d *LD_50_, lethal dose 50*.

### Molecular Docking Study Against the SIRT1

**Figure 8:**
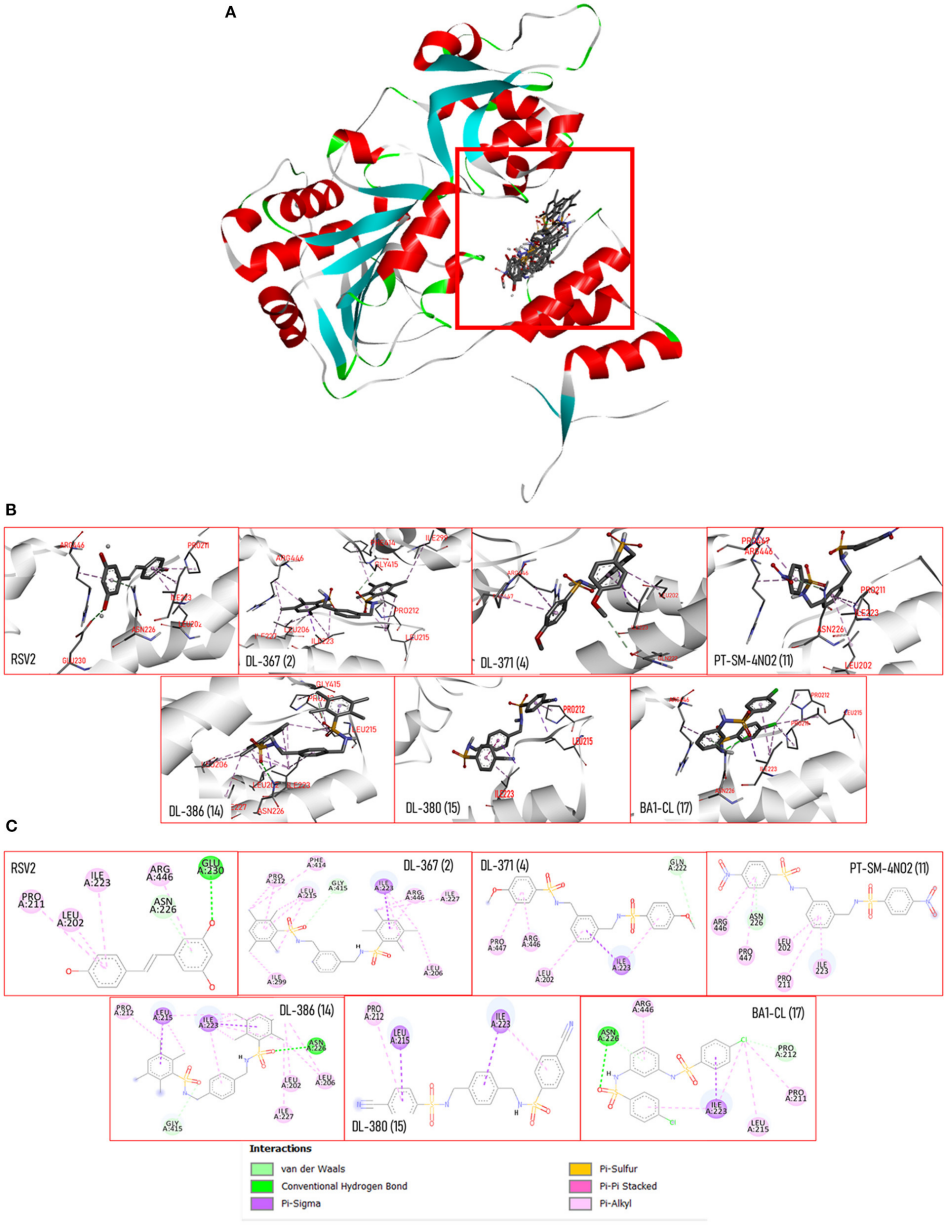
**(A)** 3D views of the crystal structure of SIRT-1 (PDB: 5BTR) with resveratrol and bis-sulfonamides showing the location of the binding sites. **(B)** 3D views of SIRT-1 binding interactions with resveratrol and bis-sulfonamides in activator binding site. **(C)** The 2D protein–ligand interaction diagram of resveratrol and bis-sulfonamides in SIRT-1 activator binding site. The interacting residues are shown as colored circles depending on the type of interaction.

Molecular docking was performed to predict possible binding modes of the investigated compounds against the target protein, SIRT1 (PDB code 5BTR). Initially, the docking protocol was validated by redocking of the co-crystallized ligand, resveratrol. The result of redocking provided an RMSD value <2.0 that indicated the reliability of the protocol for further simulations of the studied compounds (i.e., **2**, **4**, **11**, **14**, **15**, and **17**). Several runs of compound docking simulations were performed, but only a model with the highest number in a cluster of each compound was selected. The results showed that all investigated compounds could occupy within the same activator-binding site of the SIRT1 protein similar to the resveratrol molecules (RSV1 and RS2), as shown in Figure 8A. The calculated binding free energy of RSV1 and RSV2 by self-docking was −7.45 and −7.55 kcal/mol, respectively. Accordingly, the RSV2 with lower binding energy was selected as a reference. Docking results of the investigated compounds showed that all compounds provided lower binding free energy values (**2** = −11.53, **4** = −11.68, **11** = −12.08, **14** = −11.40, **15** = −10.74, and **17** = −12.61 kcal/mol) than that of the resveratrol cluster 2 (RSV2 = −7.55 kcal/mol).

Docking poses of each compound (Figure 8B) and two-dimensional ligand–protein interaction diagrams were generated to elucidate key interactions contributing to the binding of the compounds against the SIRT1 (Figure 8C). It was shown that the binding mode of the resveratrol was mainly mediated by the pi-alkyl interactions (PRO211, LEU202, ILE223, and ARG446) along with a van der Waal force (i.e., ASN226) and hydrogen bonding (i.e., GLU230). Similarly, the binding modes of the investigated compounds were mainly mediated by pi-type interactions (i.e., pi-alkyl and pi-sigma) and van der Waals interactions. In addition, these compounds shared some common interacting amino acid residues with those of the RSV2 (i.e., LEU202, ILE223, ASN226, and ARN446) that suggested their similar binding modes to that of the resveratrol. Unlike the resveratrol, hydrogen-bonding interaction with the residue GLU230 was noted from all investigated compounds, but it was revealed that two compounds (i.e., **14** and **17**) could form hydrogen interaction with ASN226 *via* their sulfonyl oxygen atoms.

## Discussion

The PD has been considered one of the global health problems in a current upcoming aging society. The management of the PD is challenging in which most of the clinically available drugs are only symptomatic drugs without preventive effects for delaying the disease progression and consequences (Hurtig, 1997; Dorsey et al., 2018). Nowadays, most of the therapeutic agents for PD treatment, both currently available and under developed ones, are those targeting the dopaminergic motor symptoms. Unfortunately, current research indicates that these therapies are intrusive and results in unexpected side effects (Sarkar et al., 2016). As a result, neuroprotection in the early stages should be focused on preventing harm or slowing the advancement of an ongoing disease. Currently, the discovery of novel neuroprotective agents for PD is one of the areas of continual interest. Despite the fact that several prospective medicines have been demonstrated in preclinical trials, no drug has been licensed as neuroprotective agents in PD (Sarkar et al., 2016). Challenges for neuroprotection in PD may be caused by a complicated damaging cascade occurring within neurons, including misfolded protein, mitochondrial dysfunction, oxidative damage, and cell death (Salamon et al., 2020). Therefore, one of the prospective molecules for PD protection was the exploration of multifunctional neuroprotective agents.

The common underlying process that leads to cellular dysfunction in PD is assumed to be oxidative stress, which is associated with mitochondrial dysfunction and neuroinflammation (Hwang, 2013). Regarding the destructive roles of the oxidative stress condition, the compounds with antioxidative effects have gained considerable attention for their neuroprotective potentials (Emerit et al., 2004; Aryal et al., 2020). Therefore, bis-sulfonamides, which were reported as a multibiological action, including antioxidant and, anti-acetylcholinesterase activities (Göçer et al., 2013; Kołaczek et al., 2014; Apaydin et al., 2019; Gök et al., 2021), as well as aromatase inhibitor (Leechaisit et al., 2019), could potentially exhibit neuroprotective effects. In this study, a set of 17 bis-sulfonamide derivatives (**1**–**17**) was explored to reveal their neuroprotective potentials in SH-SY5Y cell lines. The neuroprotective effect of sulfonamide derivatives on human neuroblastoma cell lines, SH-SY5Y, has been rarely reported in the literature. However, results from the MTT assay indicated that all tested bis-sulfonamides (**1**–**17**) are non-cytotoxic against the SH-SY5Y cell lines at all tested concentrations (1–10 μM) as demonstrated by the lack of changes in cell viability and cell morphology after the cells were treated with the compounds for 24 h when compared to the untreated control group.

Currently, 6-OHDA is commonly used as a neurotoxic agent to mimic the PD model due to its selective destruction of dopaminergic neuron. Because of structural similarity to endogenous catecholamines, 6-OHDA is recognized and uptaken by DA or noradrenaline membrane transporters. When 6-OHDA is absorbed and accumulated inside neurons, it undergoes both enzymatic breakdowns by MAO-A and autoxidation, resulting in the formation of multiple cytotoxic molecules that cause neuronal death through the production of oxidative damage and mitochondrial dysfunction (Glinka et al., 1997; Soto-Otero et al., 2000; Bové et al., 2005; Hernandez-Baltazar et al., 2017). This study revealed that exposure to 100 μM 6-OHDA could cause cell death, raise intracellular ROS levels, and induce LDH leakage. The findings are consistent with the study of Jing et al. (2015). Furthermore, this study also discovered that 6-OHDA administration diminished MMP and SIRT-1 activity.

Interestingly, pretreatment with 5 μM of six compounds out of seventeen, including DL-367 (**2**), DL-371 (**4**), PT-SM-4NO2 (**11**), DL-386 (**14**), DL-380 (**15**), and BA1-CL (**17**), before 6-OHDA exposure could improve cell viability, as shown in Figure 1. Therefore, 5 μM of each compound showing a protective effect was selected for subsequence investigation on neuroprotective effect against 6-OHDA-induced neuronal cell death (i.e., cell morphology, cell injury, ROS production, mitochondrial function, SIRT-1 activity, molecular docking, and pharmacokinetic profile prediction). The six derivatives exhibited a neuroprotective effect against the 6-OHDA-induced cellular damages on human neuronal cells. This was ascertained by a cell morphological alteration (Figure 3), reduction of LDH leakage from membrane rupture (Figure 4), and a decrease in intracellular ROS production (Figure 5). This suggests the protective potential of the compounds against 6-OHDA-induced oxidative stress. Subsequently, pretreatment with all selected compounds (**2**, **4**, **11**, **14**, **15**, and **17**) also maintained MMP, suggesting that bis-sulfonamide derivatives could significantly protect the mitochondrial dysfunction in SH-SY5Y cells.

In addition, the SIRT-1 activity assay was performed to further elucidate the underlying mechanism of the neuroprotective properties. SIRT-1, also known as a NAD-dependent deacetylase SIRT-1, is a protein encoded by the SIRT1 gene, a member of the sirtuin family. SIRT-1 protein is well-recognized for its roles in expanding longevity, antioxidant defense, and improving cell survival (Wang et al., 2010). As regards, the maintenance of SIRT-1 activity is noted for cellular protection and has gained a continual interest in neuroprotection. Upregulation of the SIRT1 or activation of the SIRT-1 activity has been noted for the enhanced cellular protection against many age-related diseases, including neurodegenerative diseases, i.e., PD (Rahman and Islam, 2011; Elibol and Kilic, 2018). Several lines of evidence on SIRT1 protein have been reported that SIRT1 acts as an upstream regulator on several transcription factors, which affect ROS generation, apoptosis, and mitochondrial dynamic (Olmos et al., 2013; Xu et al., 2018). Herein, the findings demonstrated that the pretreatment of the selected bis-sulfonamides could enhance SIRT-1 activity in the 6-OHDA-induced damage in neuronal cells. Therefore, the activation of SIRT-1 activity could possibly be one of the promising mechanisms for the neuroprotection. It is in accordance with a recent study representing that upregulation of SIRT1–FoxO3a signaling diminished Aβ-induced cytotoxicity (Lin et al., 2015) and H_2_O_2_-induced oxidative stress (Gay et al., 2018) through stimulation of the antioxidant defense system.

Moreover, the efficiency of selected compounds (i.e., **2**, **4**, **11**, **14**, **15**, and **17**) as the SIRT1 activating agent has been substantiated by molecular docking. Accordingly, *in silico* simulations were conducted to further investigate the binding behavior of the selected compounds as SIRT-1 activators. Molecular docking was performed against the target SIRT-1 protein co-crystalized with the resveratrol, a well-known SIRT-1 activator (PDB code: 5BTR; Borra et al., 2005; Cao et al., 2015). It was revealed that all compounds could occupy the binding site of the SIRT-1 in the same manner as that of the resveratrol, as shown in Figure 8, which suggest their competitive modes of binding. This was supported by the sharing of some common key interacting residues with those of the resveratrol. In addition, an essential role of the sulfonamide moiety on the binding was demonstrated by the participation of the sulfonyl oxygen in the key hydrogen interaction between ASN226 residue and compounds **14** and **17** as well as those of van der Waals interaction between GLY415 and the compounds **2** and **14**. It also showed that site-directed alteration of ASN226 lowers the deacetylation rate of SIRT1 in response to resveratrol stimulation, suggesting the important role of ASN226 interaction in SIRT1 activation. Moreover, all compounds provided lower binding energy values than that of the resveratrol, which could suggest that they may elicit a better SIRT-1 activating effect. Taken together, it was demonstrated that the neuroprotective effect of these bis-sulfonamides (**2**, **4**, **11**, **14**, **15**, and **17**) could be possibly, in part, due to their SIRT-1 activating effect which consequently upregulates the downstream signaling pathways leading to an enhancement of cell longevity (Elibol and Kilic, 2018).

Late state failure is a concerning issue in the current drug development. Most of the failures occur in the late stages of development pipeline due to their undesirable pharmacokinetic and toxicity profiles (Shaker et al., 2021). This difficulty was also encountered in sulfonamide derivatives, which are a pharmacophore in a variety of medicinal drugs. Many studies on sulfonamide-based compounds discovered that sulfonamide derivatives presented a variety of toxicities, particularly drug allergy and hepatotoxicity (Choquet-Kastylevsky et al., 2002; Supuran et al., 2003; Dorn et al., 2018; Ovung and Bhattacharyya, 2021). Herein, *in silico* prediction of drug-likeness and toxicities of the studied compounds were performed to reveal their possibilities to be further developed as anti-PD drugs. It was estimated that all of the selected compounds (i.e., **2**, **4**, **11**, **14**, **15**, and **17**) showed no immunotoxicity and hepatotoxicity with a high lethal dose (LD50), classified as a slight hazard. Moreover, the previous study on cytotoxicity of bis-sulfonamides (**1–17**) has also evaluated against the normal embryonic lung (MRC-5) cell line. Most analogs were non-cytotoxic (IC50 > 50 μg/ml) toward the normal cell, whereas compounds **2, 5, 7, 14, 15**, and **17** showed low cytotoxic effect (IC50 = 16.27–92.83 μM; Leechaisit et al., 2019). This suggested that the selected compounds may contain less toxicity in further clinical trial and be suitable for use as a medication. Pharmacokinetic prediction of the selected compounds also revealed good lipophilicity and intestinal absorption properties. However, some of them displayed undesirable drug-likeness (**15** and **17**) and weak BBB penetrating ability to reach the target site in the brain (**11**). The data suggested that additional structural modifications of the compounds may be required to improve the BBB permeability as well as drug-like properties.

Taken together, a set of six selected compounds demonstrated neuroprotective effects against the 6-OHDA-induced PD model. Findings demonstrated that possible underlying mechanisms of neuroprotection of these compounds could be due to their abilities to reduce intracellular ROS, maintain mitochondrial function, and enhance SIRT1 activity. However, this study only elucidates on SIRT1 activity, while downstream signaling proteins still need to be further investigated to have a deeper knowledge of the signaling cascade within the cell. Furthermore, pharmacokinetics properties were merely a prediction; *in vivo* and clinical studies are still needed to better elucidate their pharmacokinetic properties and mechanisms of action.

## Conclusion

Six sulfonamide derivatives, including **2**, **4**, **11**, **14**, **15**, and **17** exhibited neuroprotective effect against neuronal cell death *via* several ways including decreasing the intracellular oxidative stress, preventing mitochondrial dysfunction, modulating the SIRT1 activity, as well as minimizing the cellular morphology changes. Molecular docking also supported that these compounds may act as SIRT1 activators. Additionally, the pharmacokinetic and toxicity profiles prediction revealed that they are relatively safe and possess good bioavailability. All in all, this set of bis-sulfonamides could be a promissing compound class for further development as neuroprotective SIRT1 activators against the PD. Insight finding regarding the binding modes and key chemical features would also benefit for future design and discovery of the related compounds.

## Data Availability Statement

The raw data supporting the conclusions of this article will be made available by the authors, without undue reservation.

## Author Contributions

SP, RP, KP, WR, and PJ: investigation and methodology. KP, NS, TT, and VeP: formal analysis. WS and SP: resources. WS, RP, and KP: visualization and writing—original draft. VeP, KP, SP, and ViP: writing—review and editing. KP, RP, ViP, and SP: funding acquisition. All authors contributed to the article and approved the submitted version.

## Funding

This project was financially supported by Srinakharinwirot University and National Science, Research and Innovation Fund (NSRF) (Grant No. 026/2564), National Research Council of Thailand (NRCT), and Mahidol University (Basic Research Fund: fiscal year 2022).

## Conflict of Interest

The authors declare that the research was conducted in the absence of any commercial or financial relationships that could be construed as a potential conflict of interest.

## Publisher's Note

All claims expressed in this article are solely those of the authors and do not necessarily represent those of their affiliated organizations, or those of the publisher, the editors and the reviewers. Any product that may be evaluated in this article, or claim that may be made by its manufacturer, is not guaranteed or endorsed by the publisher.

## References

[B1] Albers D. S. Beal M. F. (2000). Mitochondrial dysfunction and oxidative stress in aging and neurodegenerative disease. Adv. Dement. Res. 2000, 133–154. 10.1007/978-3-7091-6781-6_16 10961426

[B2] Alsughayer A. Elassar A. Z. A. Mustafa S. Al Sagheer F. (2011). Nanobiotechnology. Synthesis, structure analysis and antibacterial activity of new potent sulfonamide derivatives. J. Biomater. Nanobiotechnol. 2, 143. 10.4236/jbnb.2011.22018

[B3] Alzheimer's A. (2017). Dementia. 2017 Alzheimer's disease facts and figures. Alzheimer's Dementia. 13, 325–73. 10.1016/j.jalz.2017.02.001

[B4] Apaydin S. Török M. J. B. Letters M. C. (2019). Sulfonamide derivatives as multi-target agents for complex diseases. Bioorg. Med. Chem. Lett. 29, 2042–2050. 10.1016/j.bmcl.2019.06.041 31272793

[B5] Aryal S. Skinner T. Bridges B. Weber J. T. (2020). The pathology of Parkinson's disease and potential benefit of dietary polyphenols. Molecules 25, 4382. 10.3390/molecules25194382 32987656 PMC7582699

[B6] Baker D. J. Petersen R. C. (2018). Cellular senescence in brain aging and neurodegenerative diseases: evidence and perspectives. J. Clin. Investig. 128, 1208–1216. 10.1172/JCI95145 29457783 PMC5873891

[B7] Borra M. T. Smith B. C. Denu J. M. (2005). Mechanism of human SIRT1 activation by resveratrol. J. Biol. Chem. 280, 17187–17195. 10.1074/jbc.M501250200 15749705

[B8] Bose A. Beal M. F. (2016). Mitochondrial dysfunction in Parkinson's disease. J. Neurochem. 139, 216–231. 10.1111/jnc.13731 27546335

[B9] Bové J. Prou D. Perier C. Przedborski S. (2005). Toxin-induced models of Parkinson's disease. NeuroRx 2, 484–494. 10.1602/neurorx.2.3.484 16389312 PMC1144492

[B10] Brunelle J. K. Letai A. (2009). Control of mitochondrial apoptosis by the Bcl-2 family. J. Cell Sci. 122, 437–441. 10.1242/jcs.031682 19193868 PMC2714431

[B11] Cao D. Wang M. Qiu X. Liu D. Jiang H. Yang N. (2015). Structural basis for allosteric, substrate-dependent stimulation of SIRT1 activity by resveratrol. Genes Dev. 29, 1316–1325. 10.1101/gad.265462.115 26109052 PMC4495401

[B12] Cenini G. Lloret A. Cascella R. J. O. M. Longevity C. (2019). Oxidative stress in neurodegenerative diseases: from a mitochondrial point of view. Oxid. Med. Cell. Long. 2019, 2105607. 10.1155/2019/2105607 31210837 PMC6532273

[B13] Choquet-Kastylevsky G. Vial T. Descotes J. (2002). Allergic adverse reactions to sulfonamides. Curr. Allergy Asthma Rep. 2, 16–25. 10.1007/s11882-002-0033-y 11895621

[B14] De Lau L. M. Breteler M. M. (2006). Epidemiology of Parkinson's disease. Lancet Neurol. 5, 525–535. 10.1016/S1474-4422(06)70471-9 16713924

[B15] Dorn J. M. Alpern M. McNulty C. Volcheck G. W. (2018). Sulfonamide drug allergy. Curr. Allergy Asthma Rep. 18, 1–10. 10.1007/s11882-018-0791-9 29876667

[B16] Dorsey E. Sherer T. Okun M. S. Bloem B. R. (2018). The emerging evidence of the Parkinson pandemic. J. Parkinson's Dis. 8, S3–S8. 10.3233/JPD-181474 30584159 PMC6311367

[B17] Elibol B. Kilic U. (2018). High levels of SIRT1 expression as a protective mechanism against disease-related conditions. Front. Endocrinol. 9, 614. 10.3389/fendo.2018.00614 30374331 PMC6196295

[B18] Emerit J. Edeas M. Bricaire F. (2004). Neurodegenerative diseases and oxidative stress. Biomed. Pharmacother. 58, 39–46. 10.1016/j.biopha.2003.11.004 14739060

[B19] Ferlini C. Scambia G. (2007). Assay for apoptosis using the mitochondrial probes, Rhodamine123 and 10-N-nonyl acridine orange. Nat. Protocols 2, 3111–3114. 10.1038/nprot.2007.397 18079710

[B20] Gao H. M. Hong J- S. (2008). Why neurodegenerative diseases are progressive: uncontrolled inflammation drives disease progression. Trends Immunol. 29, 357–365. 10.1016/j.it.2008.05.002 18599350 PMC4794280

[B21] Gay N. H. Phopin K. Suwanjang W. Songtawee N. Ruankham W. Wongchitrat P. (2018). Neuroprotective effects of phenolic and carboxylic acids on oxidative stress-induced toxicity in human neuroblastoma SH-SY5Y cells. Neurochem. Res. 43, 619–636. 10.1007/s11064-017-2463-x 29417471

[B22] Glinka Y. Gassen M. Youdim M. (1997). Mechanism of 6-hydroxydopamine neurotoxicity. Adv. Res. Neurodegen. 1997, 55–66. 10.1007/978-3-7091-6842-4_7 9120425

[B23] Göçer H. Akincioglu A. Öztaşkin N. Göksu S. Gülçin I. (2013). Synthesis, antioxidant, and antiacetylcholinesterase activities of sulfonamide derivatives of dopamine-related compounds. Archiv. Pharmazie. 346, 783–792. 10.1002/ardp.201300228 24591156

[B24] Gök N. Akincioglu A. Erümit Binici E. Akincioglu H. Kilinç N. Göksu S. (2021). Synthesis of novel sulfonamides with anti-Alzheimer and antioxidant capacities. Archiv. Pharmazie. 2021, e2000496. 10.1002/ardp.202000496 33749025

[B25] Hernandez-Baltazar D. Zavala-Flores L. Villanueva-Olivo A. (2017). The 6-hydroxydopamine model and parkinsonian pathophysiology: novel findings in an older model. Neurología 32, 533–539. 10.1016/j.nrl.2015.06.011 26304655

[B26] Hurtig H. I. (1997). Problems with current pharmacologic treatment of Parkinson's disease. Exp. Neurol. 144, 10–16. 10.1006/exnr.1996.6380 9126144

[B27] Hwang O. (2013). Role of oxidative stress in Parkinson's disease. Exp. Neurobiol. 22”11. 10.5607/en.2013.22.1.11 23585717 PMC3620453

[B28] Jing X. Shi H. Zhang C. Ren M. Han M. Wei X. (2015). Dimethyl fumarate attenuates 6-OHDA-induced neurotoxicity in SH-SY5Y cells and in animal model of Parkinson's disease by enhancing Nrf2 activity. Neuroscience 286, 131–140. 10.1016/j.neuroscience.2014.11.047 25449120

[B29] Kalia L. V. Lang A. E. (2015). Parkinson's disease. Lancet 386, 896–912. 10.1016/S0140-6736(14)61393-3 25904081

[B30] Kanasi E. Ayilavarapu S. Jones J. (2016). The aging population: demographics and the biology of aging. Periodontology 72, 13–18. 10.1111/prd.12126 27501488

[B31] Kołaczek A. Fusiarz I. Ławecka J. Branowska D. (2014). Biological activity and synthesis of sulfonamide derivatives: a brief review. Chemik 68, 620–628. 32888265

[B32] Lee K. H. Cha M. Lee B. H. (2020). Neuroprotective effect of antioxidants in the brain. Int. J. Mol. Sci. 21, 7152. 10.3390/ijms21197152 32998277 PMC7582347

[B33] Leechaisit R. Pingaew R. Prachayasittikul V. Worachartcheewan A. Prachayasittikul S. Ruchirawat S. (2019). Synthesis, molecular docking, and QSAR study of bis-sulfonamide derivatives as potential aromatase inhibitors. Bioorg. Med. Chem. 27”115040. 10.1016/j.bmc.2019.08.001 31416738

[B34] Lin C. L. Huang W-, N. Huang H. Li H.. (2015). Hydrogen-rich water attenuates amyloid β-induced cytotoxicity through upregulation of Sirt1-FoxO3a by stimulation of AMP-activated protein kinase in SK-N-MC cells. Chemico-Biol. Interact. 240, 12–21. 10.1016/j.cbi.2015.07.013 26271894

[B35] Lynch T. Price A. (2007). The effect of cytochrome P450 metabolism on drug response, interactions, and adverse effects. Am. Fam. Phys. 76, 391–396. 17708140

[B36] Martinou J.- C. Youle R. (2011). Mitochondria in apoptosis: Bcl-2 family members and mitochondrial dynamics. Dev. Cell 21, 92–101. 10.1016/j.devcel.2011.06.017 21763611 PMC3156409

[B37] Morris G. M. Goodsell D. S. Halliday R. S. Huey R. Hart W. E. Belew R. K. (1998). Automated docking using a Lamarckian genetic algorithm and an empirical binding free energy function. J. Comput. Chem. 19, 1639–62. 10.1002/(SICI)1096-987X(19981115)19:14<1639::AID-JCC10>3.0.CO;2-B

[B38] Morris G. M. Huey R. Lindstrom W. Sanner M. F. Belew R. K. Goodsell D. S. (2009). AutoDock4 and AutoDockTools4: automated docking with selective receptor flexibility. J. Comput. Chem. 30, 2785–2791. 10.1002/jcc.21256 19399780 PMC2760638

[B39] Olmos Y. Sánchez-Gómez F. J. Wild B. García-Quintans N. Cabezudo S. Lamas S. (2013). SirT1 regulation of antioxidant genes is dependent on the formation of a FoxO3a/PGC-1α complex. Antioxid. Redox Signal. 19, 1507–1521. 10.1089/ars.2012.4713 23461683 PMC3797451

[B40] Ovung A. Bhattacharyya J. (2021). Sulfonamide drugs: structure, antibacterial property, toxicity, and biophysical interactions. Biophys. Rev. 13, 259–272. 10.1007/s12551-021-00795-9 33936318 PMC8046889

[B41] Peng X. Hu T. Zhang Y. Zhao A. Natarajan B. Wei J. (2020). Synthesis of caffeic acid sulfonamide derivatives and their protective effect against H2O2 induced oxidative damage in A549 cells. RSC Adv. 10, 9924–9933. 10.1039/D0RA00227E 35692719 PMC9122571

[B42] Potashkin J. Seidl S. E. (2011). The promise of neuroprotective agents in Parkinson's disease. Front. Neurol. 2, 68. 10.3389/fneur.2011.00068 22125548 PMC3221408

[B43] Pratiwi R. Nantasenamat C. Ruankham W. Suwanjang W. Prachayasittikul V. Prachayasittikul S. (2021). Mechanisms and neuroprotective activities of stigmasterol against oxidative stress-induced neuronal cell death *via* sirtuin family. Front. Nutr. 8, 648995. 10.3389/fnut.2021.648995 34055852 PMC8149742

[B44] Rahman S. Islam R. (2011). Mammalian Sirt1: insights on its biological functions. Cell Commun. Signal. 9, 1–8. 10.1186/1478-811X-9-11 21549004 PMC3103488

[B45] Rosenfeld C. S. Shay D. A. Vieira-Potter V. J. (2018). Cognitive effects of aromatase and possible role in memory disorders. Front. Endocrinol. 9, 610. 10.3389/fendo.2018.00610 30386297 PMC6199361

[B46] Salamon A. Zádori D. Szpisjak L. Klivényi P. Vécsei L. (2020). Neuroprotection in Parkinson's disease: facts and hopes. J. Neural Transmission 127, 821–829. 10.1007/s00702-019-02115-8 31828513 PMC7242234

[B47] Sarkar S. Raymick J. Imam S. (2016). Neuroprotective and therapeutic strategies against Parkinson's disease: recent perspectives. Int. J. Mol. Sci. 17, 904. 10.3390/ijms17060904 27338353 PMC4926438

[B48] Shaker B. Ahmad S. Lee J. Jung C. Na D. (2021). In silico methods and tools for drug discovery. Comput. Biol. Med. 137, 104851. 10.1016/j.compbiomed.2021.104851 34520990

[B49] Sooknual P. Pingaew R. Phopin K. Ruankham W. Prachayasittikul S. Ruchirawat S. (2020). Synthesis and neuroprotective effects of novel chalcone-triazole hybrids. Bioorg. Chem. 105, 104384. 10.1016/j.bioorg.2020.104384 33130346

[B50] Soto-Otero R. Méndez-Álvarez E. Hermida-Ameijeiras Á. Muñoz-Patiño A. M. Labandeira-Garcia J. L. (2000). Autoxidation and neurotoxicity of 6-hydroxydopamine in the presence of some antioxidants: potential implication in relation to the pathogenesis of Parkinson's disease. J. Neurochem. 74, 1605–1612. 10.1046/j.1471-4159.2000.0741605.x 10737618

[B51] Supuran C. T. Casini A. Scozzafava A. (2003). Protease inhibitors of the sulfonamide type: anticancer, antiinflammatory, and antiviral agents. Medicinal Res. Rev. 23, 535–558. 10.1002/med.10047 12789686

[B52] Wang J. Fivecoat H. Ho L. Pan Y. Ling E. Pasinetti G. M.. (2010). The role of Sirt1: at the crossroad between promotion of longevity and protection against Alzheimer's disease neuropathology. Biochim. Biophys. Acta 1804, 1690–1694. 10.1016/j.bbapap.2009.11.015 19945548

[B53] Winklhofer K. F. Haass C. (2010). Mitochondrial dysfunction in Parkinson's disease. Biochim. Biophys. Acta 1802, 29–44. 10.1016/j.bbadis.2009.08.013 19733240

[B54] Wood-Kaczmar A. Gandhi S. Wood N. (2006). Understanding the molecular causes of Parkinson's disease. Trends Mol. Med. 12, 521–528. 10.1016/j.molmed.2006.09.007 17027339

[B55] Wu F. Zhou Y. Shen L. Li L. Chen X. Wang G. X.. (2020). Computational approaches in preclinical studies on drug discovery and development. Front. Chem. 8, 726. 10.3389/fchem.2020.00726 33062633 PMC7517894

[B56] Xu J. Jackson C. W. Khoury N. Escobar I. Perez-Pinzon M. A. (2018). Brain SIRT1 mediates metabolic homeostasis and neuroprotection. Front. Endocrinol. 2018, 702. 10.3389/fendo.2018.00702 30532738 PMC6265504

[B57] Zhu H. Xia M. (2016). High-Throughput Screening Assays in Toxicology. Berlin: Springer. 10.1007/978-1-4939-6346-1

